# Retraction: Influence of chemical composition on the amount of second phases precipitates and transformation temperatures of TiNiPdCu shape memory alloys prepared through novel powder metallurgy route

**DOI:** 10.1039/d4ra90054e

**Published:** 2024-05-15

**Authors:** Abid Hussain, Afzal Khan, Muhammad Imran Khan, Saif Ur Rehman

**Affiliations:** a Department of Mechanical Engineering, University of Engineering and Technology Peshawar Pakistan abidhussain@uetpeshawar.edu.pk; b Ghulam Ishaq Khan (GIK) Institute of Science, Engineering and Technology Swabi, Topi 23640 Khyber Pakhtonhwa Pakistan; c Institute of Industrial Control System Rawalpindi Pakistan

## Abstract

Retraction of ‘Influence of chemical composition on the amount of second phases precipitates and transformation temperatures of TiNiPdCu shape memory alloys prepared through novel powder metallurgy route’ by Abid Hussain *et al.*, *RSC Adv.*, 2023, **13**, 29376–29392, https://doi.org/10.1039/D3RA05513B.

The Royal Society of Chemistry hereby wholly retracts this *RSC Advances* article due to concerns with the reliability of the data.

The resolution of many of the SEM images is very poor, and there appear to be some repeating elements in the SEM images in Fig 7c and d. Additionally, the authors have said that the images have been combined from various sections of the same SEM image, leading to concerns that the SEM images may not be genuine. The authors have not been able to provide the original raw images for the SEM images in Fig. 1, 3, 4, 5, 6 and 7.

Given the significance of these concerns, and the lack of raw data, the findings presented in this paper are no longer reliable.

This retraction supersedes the information provided in the expression of concern related to this article.

Signed: Afzal Khan, Muhammad Imran Khan, Abid Hussain, Saif Ur Rehman

Date: 7th May 2024

Retraction endorsed by Laura Fisher, Executive Editor, *RSC Advances*

## Supplementary Material

